# Pharmacotherapy for Adverse Events Reduces the Length of Hospital Stay in Patients Admitted to Otolaryngology Ward: A Single Arm Intervention Study

**DOI:** 10.1371/journal.pone.0115879

**Published:** 2014-12-30

**Authors:** Akio Suzuki, Ryo Kobayashi, Shinji Okayasu, Bunya Kuze, Mitsuhiro Aoki, Keisuke Mizuta, Yoshinori Itoh

**Affiliations:** 1 Department of Pharmacy, Gifu University Hospital, Gifu, Japan; 2 Department of Otolaryngology, Gifu University Graduate School of Medicine, Gifu, Japan; 3 Department of Medical Information Division, Gifu University Hospital, Gifu, Japan; Central South University, China

## Abstract

**Background:**

To determine whether adverse events extend the duration of hospitalization, and to evaluate the effectiveness of medical intervention in ameliorating adverse events and reducing the prolonged hospital stay associated with adverse events.

**Methods:**

A single arm intervention study was conducted from October 2012 to March 2014 in the otolaryngology ward of a 614-bed, university-affiliated hospital. Adverse events were monitored daily by physicians, pharmacists and nurses, and recorded in the electronic medical chart for each patient. Appropriate drug management of adverse events was performed by physicians in liaison with pharmacists. The Kaplan-Meier method was used to assess the length of hospitalization of patients who underwent medical intervention for adverse events.

**Results:**

Of 571 patients admitted to the otolaryngology ward in a year, 219 patients (38.4%) experienced adverse events of grade ≥2. The duration of hospitalization was affected by the grade of adverse events, with a mean duration of hospital stay of 9.2, 17.2, 28.3 and 47.0 days for grades 0, 1, 2, and 3–4, respectively. Medical intervention lowered the incidence of grade ≥2 adverse events to 14.5%. The length of hospitalization was significantly shorter in patients who showed an improvement of adverse events after medical intervention than those who did not (26.4 days vs. 41.6 days, hazard ratio 1.687, 95% confidence interval: 1.260–2.259, P<0.001). A multivariate Cox proportional hazard analysis indicated that insomnia, constipation, nausea/vomiting, infection, non-cancer pain, oral mucositis, odynophagia and neutropenia were significant risk factors for prolongation of hospital stay.

**Conclusion:**

Patients who experienced adverse events are at high risk of prolonged hospitalization. Medical intervention for adverse events was found to be effective in reducing the length of hospital stay associated with adverse events.

## Introduction

The occurrence of adverse events in hospitalized patients is a major medical problem [1]–[4]. Adverse event is defined as an injury related to medical management and not to disease complication [5]. This definition incorporates diagnosis and treatment, failure to diagnose or treat, and the systems and equipment used to deliver care. In addition, Brennan et al. defined an adverse event as an injury that prolongs hospital stay, produces disability at the time of discharge, or both [6]. In several studies, adverse events were associated with prolonged hospital stays [7]–[9], and increase the cost of care associated with hospitalization and medical management of the adverse event. In a prospective cohort study of 11 medical and surgical units of two hospitals, Bates et al. [10] reported a total of 247 adverse drug events in 207 admissions, which were associated with an additional stay of 2.2 days and a cost increase of $3,244.

The prevention and timely remedy of adverse events in hospitalized patients is of the upmost importance in reducing healthcare costs, and thus, adverse events should be monitored by all healthcare professionals, including physicians, nurses, nutritionists, pharmacists and other medical staff. Evans et al. reported that the prevention of adverse events through computerized surveillance can reduce the length of hospitalization [11]. However, it is still unknown how adverse events have influence on the duration of hospitalization.

In the present study, we investigated the incidence of adverse events in hospitalized patients in the otolaryngology ward from October 2012 to March 2014. We evaluated the clinical outcome of adverse events managed with pharmacotherapy by a multidisciplinary team of otolaryngologists, nurses and pharmacists. We also investigated the risks for prolonged hospital stay associated with various adverse events.

## Methods

### Ethics statement

The study was conducted according to the guidelines for human studies determined by the ethical committee of Gifu University Graduate School of Medicine and the Government of Japan, and was approved by the Medical Review Board of Gifu University Graduate School of Medicine (approval no. 25-221).

### Study design

This was a single arm intervention study conducted at Gifu University Hospital, a 614-bed hospital affiliated with Gifu University. All patients, except for those whose age was under 18 year-old, admitted during a period between October 2012 and March 2014 were included in this study. Adverse events were monitored daily by pharmacists and nurses, and recorded in the electronic medical chart for each patient. During the study period, physicians and pharmacists were in charge of medical intervention in case of the occurrence of moderate or severe adverse events. Appropriate drug management of adverse events was performed by physicians in liaison with pharmacists, and the judgment whether or not the intervention improved the adverse events was conducted until the end of the treatment.

### Assessment and intervention of adverse events

Adverse event was defined harm due to medications (adverse drug event), surgery, radiation therapy, or those that occurred during the course of the disease, excluding medical errors, system errors and equipment failure, in the present study. The severity of adverse events was graded according to the Common Terminology Criteria for Adverse Events (CTCAE, National Cancer Institute, MD, USA) version 4.0, unless otherwise indicated. For constipation, the presence of hard stools or patient’s complaint about reduced frequency of defecation was regarded as grade 1, and no bowel movement continued for at least 72 hours was regarded as grade 2, obstipation that requires manual evacuation was regarded as grade 3. Odynophagia, defined as a painful swallowing induced by chemoradiotherapy, was graded as follows: no or mild symptomatic state without the need of intervention was regarded as grade 1, moderate symptom without interference of oral intake or requirement of modified diet was grade 2, severe pain with interference of oral intake was grade 3, life-threatening consequences requiring urgent intervention was grade 4. Neutropenia is the decrease in neutrophil count to 1,000 – <1,500/mm^3^ (grade 2), 500 – <1,000/mm^3^ (grade 3) and <500/mm^3^ (grade 4). Cancer pain is a chronic pain caused by the progression of cancer disease. Vomiting was induced after surgery (by opioids) or chemotherapy, or elicited by gastrointestinal disorders. Infection included catheter-related infection, lung infection, skin infection, urinary tract infection, wound infection and febrile neutropenia. Abnormal electrolytes included hypercalcemia, hyperkalemia, hypocalcemia, hypokalemia, hypomagnesemia, hyponatremia and hypophosphatemia. Gastrointestinal dysfunction was an inclusive symptom except for constipation, diarrhea, oral mucositis, nausea and vomiting. Pain was classified into cancer pain and non-cancer pain, and evaluated by numeric rate scale (NRS) in addition to the CTCAE. Interventions by clinical pharmacists were generally carried out in patients showing grade ≥2 adverse events.

The duties of the clinical pharmacists included the following: interviewing all patients on admission and documenting the medications brought into hospital and their medication history, daily review of laboratory data, verification of prescriptions, monitoring of adverse events, provision of drug information to the medical staff, and patient education. Physicians considered the necessity of medical intervention based on the recommendation by pharmacists. For the judgment of the effectiveness of the intervention to some adverse events, including nausea, pain, tumor pain, mucositis oral, peripheral neuropathy and odynophagia, patient-reporting scale such as NRS, VAS (visual analogue scale) or FS (face scale) was used. The objective assessment of infection was carried out by culturing, gram stain, diagnostic imaging, physical findings and blood test. Pharmacotherapy for adverse events (including adverse drug events) was based on the clinical practice guidelines for nausea [12], tumor pain [13] and non-cancer pain [14], oral mucositis [15], infection [16], febrile neutropenia [17], and diarrhea [18]. The detail of the medication treatment was shown in Table 1. The effects of the medical intervention was evaluated during the course of hospitalization, except for chemotherapy-induced nausea and vomiting (CINV), where CINV was monitored during 5 days after the start of chemotherapy.

**Table 1:** Contents of madical intervention for each grade>2 adverse events with highest incidence in 18 months.

Adverse events intervened	Interventions
Insomnia	brotizolam (24), zolpidem (16), flunitrazepam (8), alprazolam (6), trazodone (5), others (14)
Constipation	snnosides (22), picosulfate Na (12), magnesium oxide (12), bisacodyl (7), glycerine (5), others (4)
Nausea/vomting	olanzapine (19), metoclopramide (15), prochlorperazine (7), domperidone (8), aprepitant (5), others (6)
Infection	Sulbactam/Ampicillin (15), Ceftriaxone (7), Tazobactam/Piperacillin (6), Meropenem (4) others (14)
Non-cancer pain	loxoprofen (24), tramadol (11), acetaminophen (6), pentazocine (3), celecoxib (2), others (2)
Electrolytes	correction of serum Ca (15), Na (11), K (4), and phosphorus (1)
Oral mucositis	steroids (14), local anesthetics (10), loxoprofen (5), acetaminophen (4), tramadol (3), others (6)
Neutrophenia	G-CSF (19), follow-up (7)
Odynophagia	tramadol (8), loxoprofen (7), acetaminophen (7), local anesthetics (5), opioids (3)
Cancer pain	opioids (9), tramadol (7), acetaminophen (4), pregabalin (4), loxoprofen (4)
Gastrointestinal dysfunction	proton pump inhibitors (8), H_2_ blockers (5)
Anemia	follow-up (7), blood transfusion (3), sodium ferrous citrate (3)
Dermatitis radiation	steroids (11), azulene (1)
Delirium	risperidone (4), haloperidol (3), quetiapine (2), olanzapine (1), alprazolam (1), ethyl loflazepate (1)
ALT increased	glycyrrhizin (5), change or cessation of drugs (4), follow-up (2)
Hypertension	calcium channel blocker (5), angiotensin receptor antagonist (3), angiotensin-converting enzyme inhibitor (1)
Diarrhea	albumin tannate (4), antiflatulent (3), loperamide (2)

### Data analysis

The following patient data were recorded in specially prepared Microsoft Excel 2010 (Microsoft Corp. Redmond, WA, USA) spreadsheets: patient age and sex; date of admission and discharge; diagnosis; purpose of hospitalization; list of private medications; pharmacists’ prescription proposals; and adverse events, their grade, and outcome of intervention. The duration of hospital stay was documented in Kaplan-Meier plots and the mean hospital stay was statistically compared using the Mantel-Cox log rank test. Univariate and multivariate Cox proportional hazard analyses were carried out to determine the risk for prolongation of hospital stay associated with adverse events. Data were analyzed using SPSS version 11 (SPSS Inc., Chicago, IL, USA) and GraphPad Prism version 6.0 (GraphPad Software, San Diego, CA, USA). The incidence of adverse events was statistically analyzed using McNemar’s test for paired non-parametric variables. P-values of <0.05 was considered statistically significant.

## Results

### Patient demographics

A total of 640 patients were admitted into the otolaryngology ward of the Gifu University Hospital during one year from October 2012 to April 2014. Among them, 69 patients of less than 18 year-old in age were excluded from the study, and 571 patients [cancer patients: n = 246 (43.1%), non-cancer patients: n = 325 (56.9%)] were the subjects of the present study. Patient demographics were shown in Table 2. The mean age was 58.0 years (10–90 percentiles, 31–76 year-old) and the mean duration of hospital stay was 22.1 days (10–90 percentiles, 4.0–57.9 days). Of 246 cancer patients, 125 (50.8%) and 121 patients (49.2%) were hospitalized for the purpose of surgery and chemoradiotherapy, respectively. On the other hand, of 325 non-cancer patients, 238 (73.2%), 66 (20.3%) and 21 patients (6.6%) were hospitalized to undergo surgery, pharmacotherapy and examination, respectively. The most frequent type of cancer was hypopharyngeal cancer (17.5%), followed by oropharyngeal cancer (16.7%), laryngeal cancer (15.9%), nasal and paranasal cancer (14.6%), thyroid gland cancer (13.0%) and lip and oral cavity cancer (6.9%). In non-cancer patients, the most common disease was head and neck benign neoplasm (20.0%), followed by sinusitis (10.5%), otitis media (9.2%), hypacusia (7.7%), cicatricial contracture (7.4%) and tonsillitis (5.2%).

**Table 2:** Patient demographics.

No. of patients (male/female)	571 (342/229)
Age (mean, 10–90^th^ percentiles)	58.0 (31–76)
Length of hospital stay (mean day, 10–90^th^ percentiles)	22.1 (4.0–57.9)
Objective of hospitalization (No. of patients)	
Cancer patients	
Surgery	125 (50.8%)
Radiochemotherapy	121 (49.2%)
Non-cancer patients	
Surgery	238 (73.2%)
Pharmacotherapy	66 (20.3%)
Investigation	21 (6.6%)
Disease (No. of patients, %)	
Cancer patients	
Hypopharyngeal cancer	43 (17.5%)
Oropharyngeal cancer	41 (16.7%)
Laryngeal cancer	39 (15.9%)
Nasal and paranasal cancer	36 (14.6%)
Thyroid gland cancer	32 (13.0%)
Lip and oral cavity cancer	17 (6.9%)
Others	38 (15.4%)
Non-cancer patients	
Head and neck neoplasm	65 (20.0%)
Sinusitis	34 (10.5%)
Otitis media	30 (9.2%)
Hypacusia	25 (7.7%)
Cicatricial contracture	24 (7.4%)
Tonsillitis	17 (5.2%)
Others	130 (40.0%)

### Incidence of adverse events and the effect of pharmacotherapy

**Figure 1:**
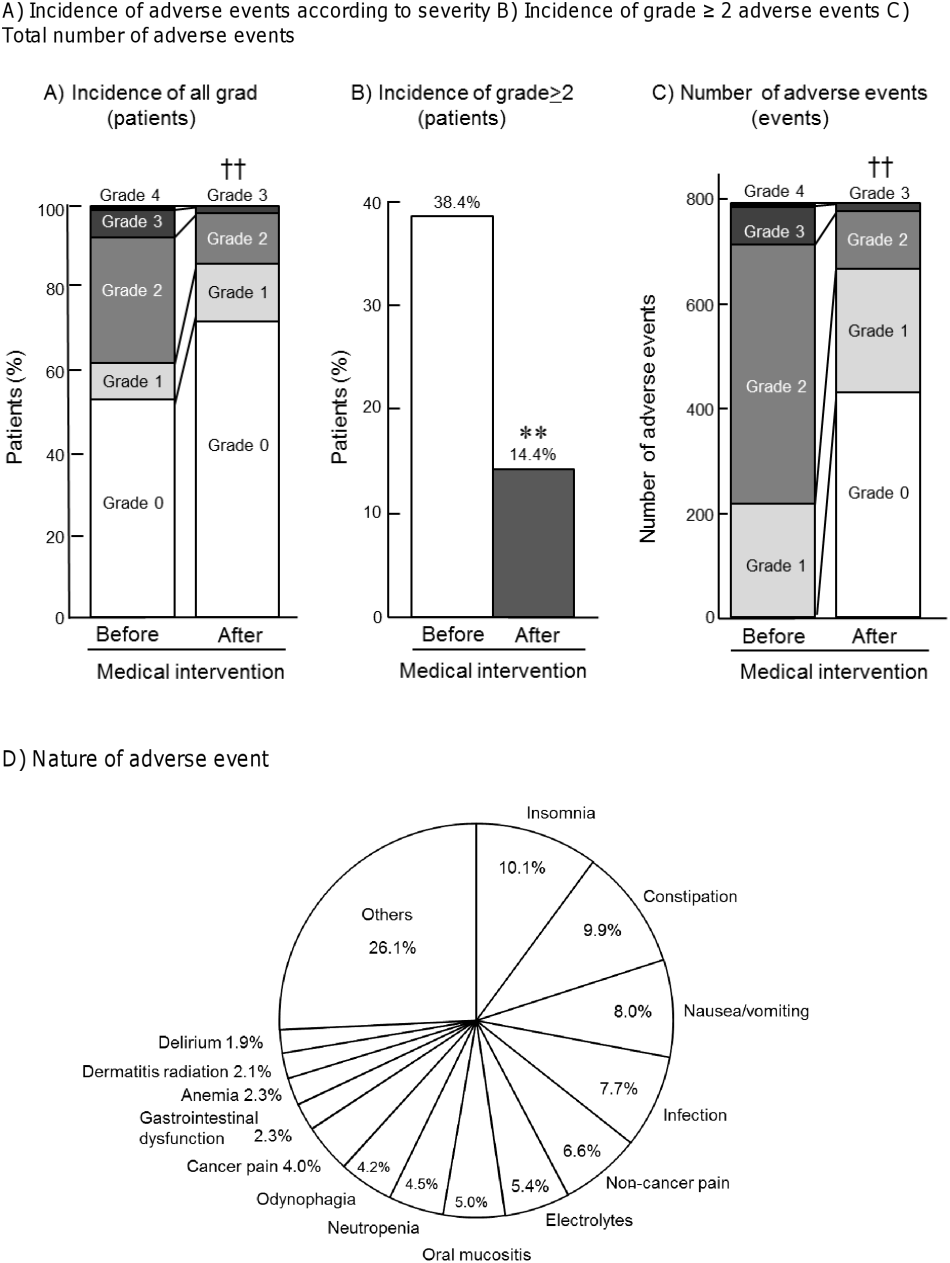
Effect of medical intervention on the incidence of (A) all four grades of adverse events, (B) incidence of grade ≥2 adverse events, and (C) total number of adverse events, (D) The types of adverse events in hospitalized patients in the otolaryngology ward Wilcoxon signed rank test was used for statistical comparisons in (A) and (C), while McNemar’s test was used to analyze data in (B). ††P<0.001 by Wilcoxon singed rank test, **P<0.001 by McNemar test.

In all patients, the incidence rates of grade ≥1 and grade ≥2 adverse events were 47.5% and 38.4%, respectively (Fig. 1A, B). The total number of adverse events was 789, in which 27.1%, 62.9%, 8.7%, and 1.3% were grade 1, 2, 3, and 4, respectively (Fig. 1C). After medical intervention, the incidence and the number of adverse events were significantly reduced: the incidence rates of grade ≥1 and grade ≥2 events were 28.4% (P<0.01, Fig. 1A) and 14.4% (P<0.01, Fig. 1B), respectively, while the total number of adverse events was 358 events (P<0.01, Fig. 1C). The most common adverse event was insomnia (10.1%), followed by constipation (9.9%), nausea/vomiting (8.0%), infection (7.7%), non-cancer pain (6.6%), abnormal electrolytes (5.4%), oral mucositis (5.0%), neutropenia (4.5%), odynophagia (4.2%), cancer pain (4.0%), gastrointestinal dysfunction (2.3%), anemia (2.3%), radiotherapy-induced dermatitis (2.1%) and delirium (1.9%) (Fig. 1D).

### Influence of adverse events of various grades on hospital stay

**Figure 2:**
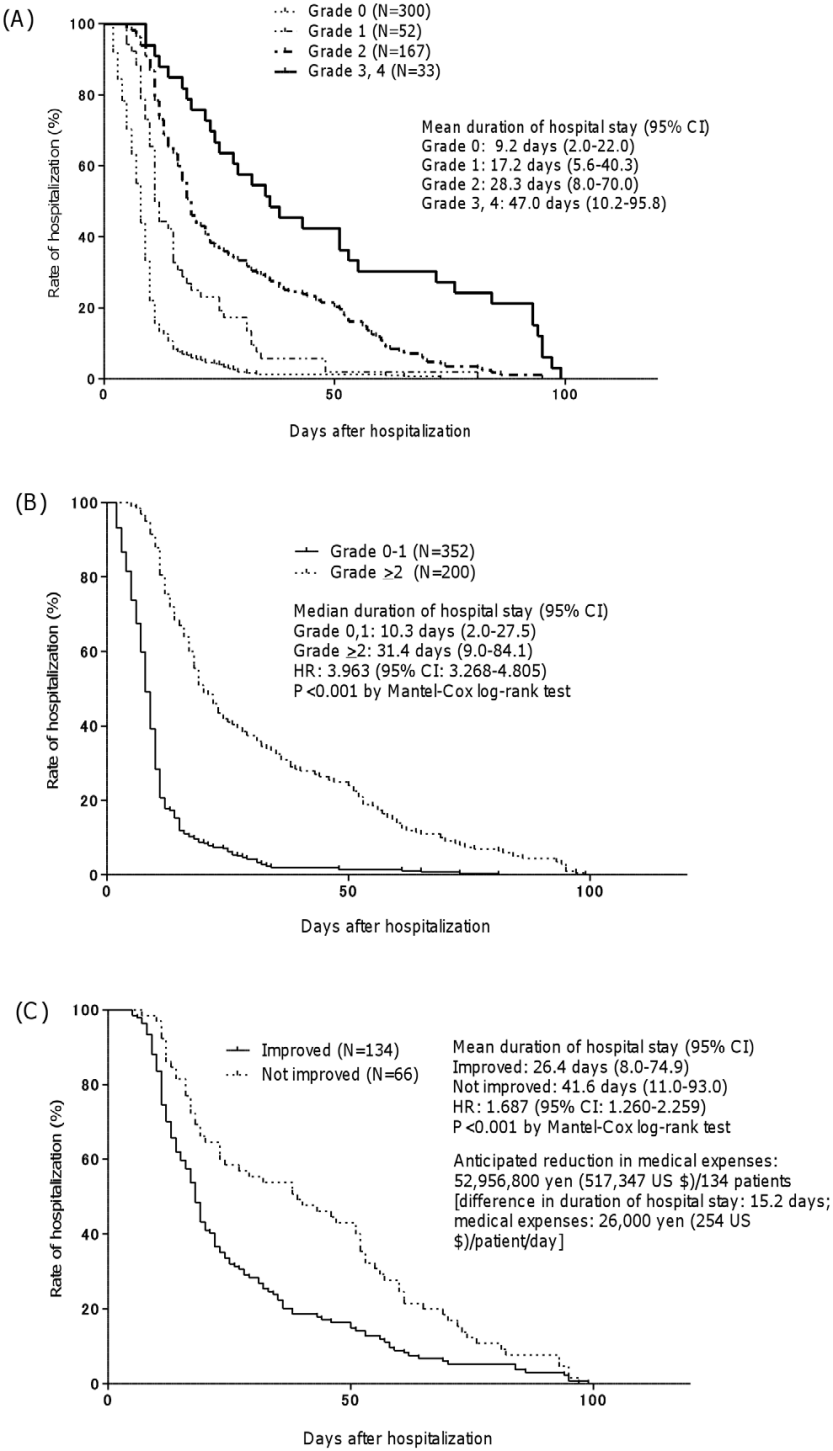
Kaplan-Meier plots showing the duration of hospital stay of patients (A) with adverse events according to grade, (B) with either grade <2 or grade ≥2 adverse events, and (C) who responded to medical intervention. Data were statistically compared using Mantel-Cox log rank test.

As a whole, mean duration of hospital stay was 9.2 days [95% confidence interval (CI) 2.0–22.0 days, N = 300] for grade 0, 17.2 days (95% CI 5.6–40.3 days, N = 52) for grade 1, 28.3 days (95% CI 8.0–70.0 days, N = 167) for grade 2, and 47.0 days (95% CI 10.2–95.8 days, N = 33) for grades ≥3 (Fig. 2A). The mean length of hospitalization for patients with grade <2 events was significantly shorter than those with grade ≥2 events (10.3 vs 31.4, hazard ratio 3.963, 95% CI 3.268–4.805, P<0.001) (Fig. 2B). The mean duration of hospital stay of patients who showed an improvement of adverse events (to grade 0 or 1) after medical intervention was significantly shorter than those who did not (26.4 days vs. 41.6 days, hazard ratio 1.687, 95% CI 1.260–2.259, P<0.001 by Mantel-Cox log rank test) (Fig. 2C). On the assumption that the average cost of the Diagnosis Procedure Combination (DPC) for hospitalization per day is 26,000 Japanese yen (equivalent to USD 254), the mean reduction of hospital stay was 15.2 days, and the number of patients who showed improvement of adverse events by medical intervention was 134, the cost saving in the reduction of hospital stay was estimated to be 53.0 million Japanese yen (USD 517,000) during 18 months.

**Figure 3:**
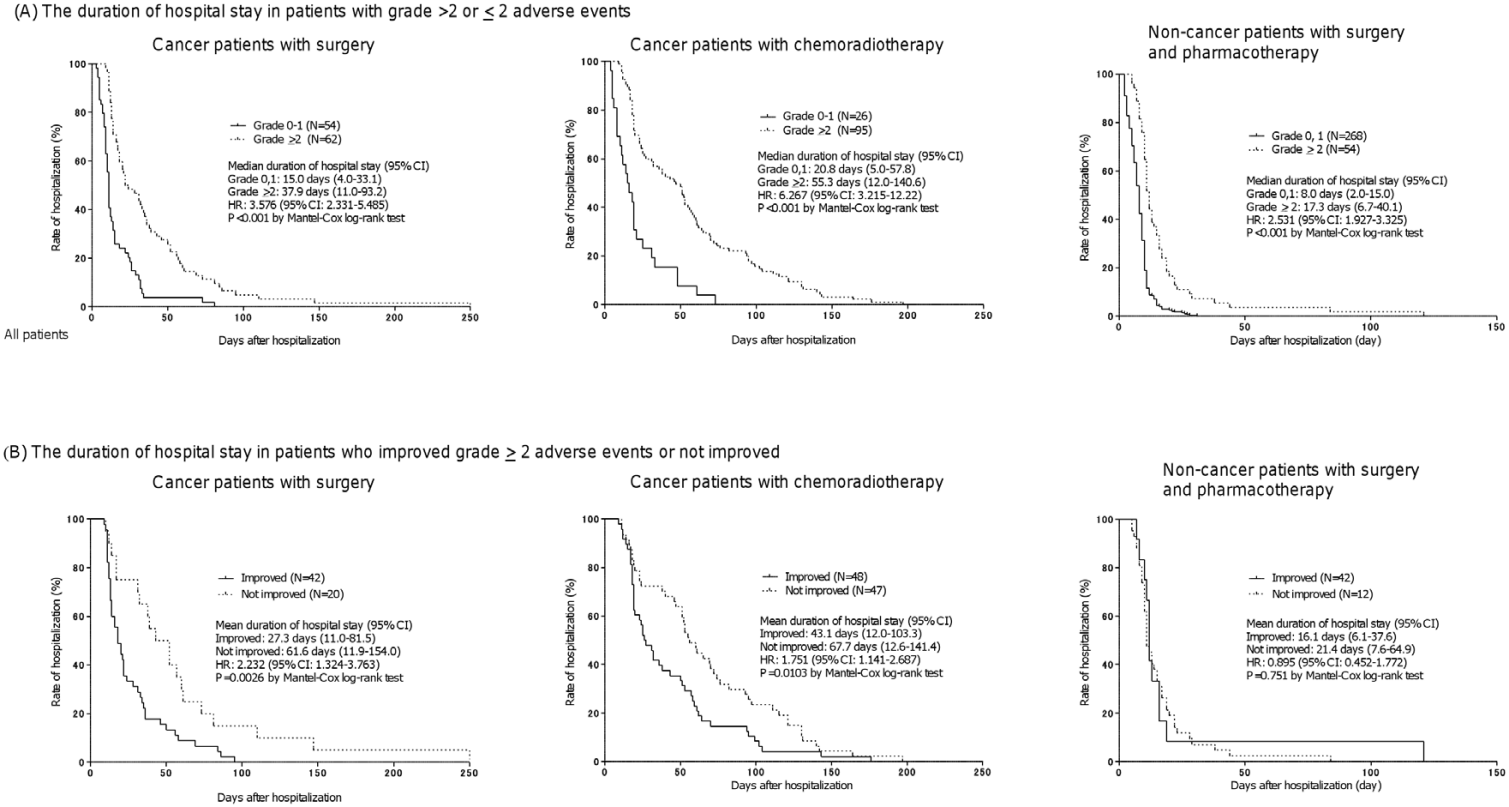
Kaplan-Meier plots showing the duration of hospital stay (A) with either grade <2 or grade ≥2 adverse events in cancer patients with operation, cancer patients with radiochemotherapy or non-cancer patients with surgery and pharmacotherapy and (B) who responded to medical intervention in cancer patients with operation, cancer patients with radiochemotherapy or non-cancer patients with surgery and pharmacotherapy. Data were statistically compared using Mantel-Cox log.

Subgroup analyses indicated that similar results were obtained in cancer patients with surgery, in those with radiochemotherapy, as well as in non-cancer patients, in which the mean length of hospitalization for patients with grade <2 events was significantly shorter than those with grade ≥2 events (cancer patients with surgery: 15.0 vs 37.9, hazard ratio 3.576, 95% CI 2.331–5.485, P<0.001; cancer patients with chemoradiotherapy: 20.8 vs 55.3, hazard ratio 6.267, 95% CI 3.215–12.22, P<0.001; non-cancer patients: 8.0 vs 17.3, hazard ratio 2.531, 95% CI 1.927–3.325, P<0.001) (Fig. 3A). The mean duration of hospital stay of patients who showed improvement in the adverse events (to grade 0 or 1) after medical intervention was significantly shorter than those without improvement of the adverse events after intervention in cancer patients with surgery (27.3 vs 61.6, hazard ratio 2.232, 95% CI 1.324–3.763, P = 0.0026) and in cancer patients with chemoradiotherapy (43.1 vs 67.7, hazard ratio 1.751, 95% CI 1.141–2.687, P = 0.0103) but not in non-cancer patients (16.1 vs 21.4, hazard ratio 0.895, 95% CI 0.452–1.772, P = 0.7510) (Fig. 3B).

### Effect of pharmacotherapy on the incidence and grade of adverse events

**Figure 4:**
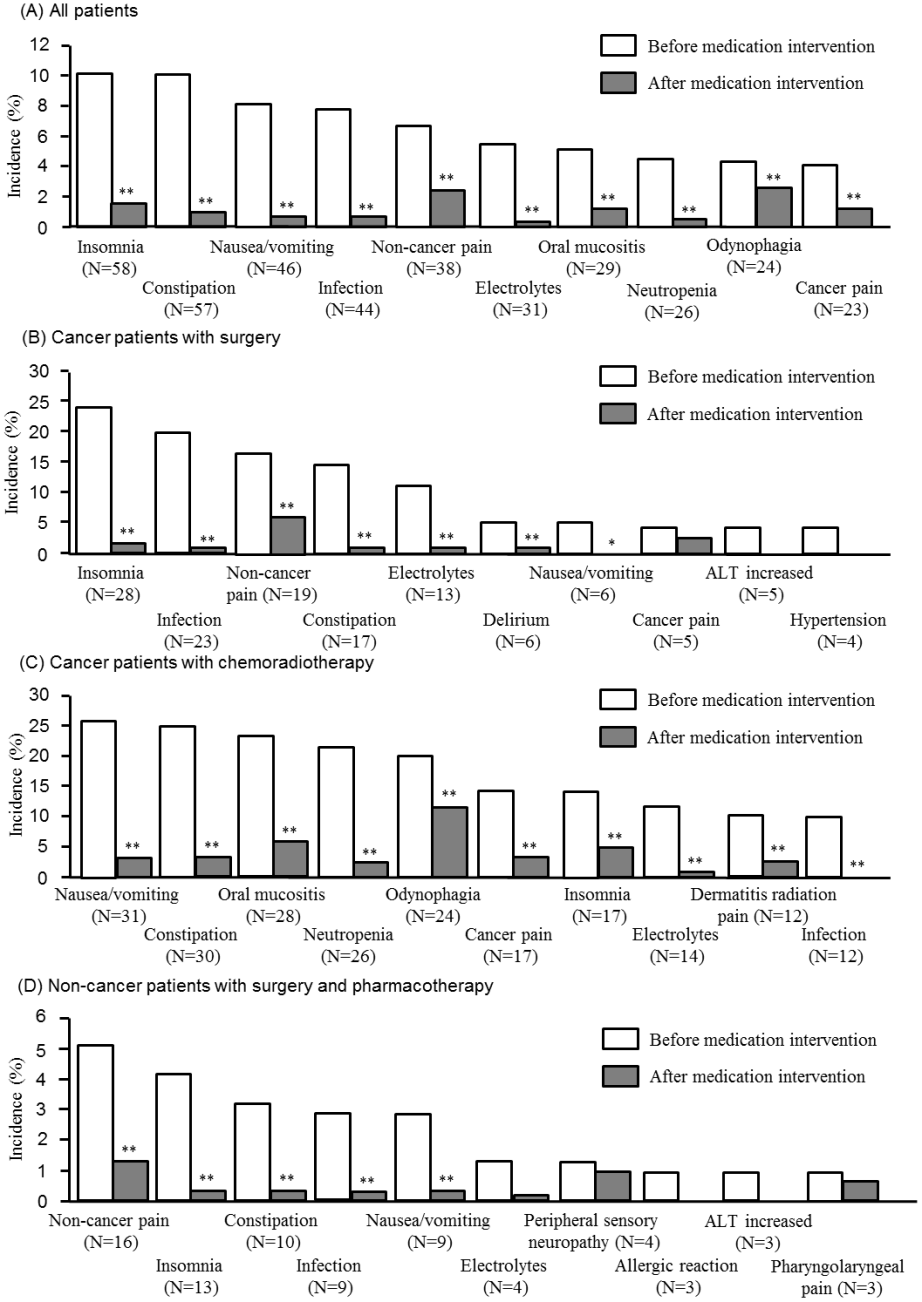
Incidence of adverse events were shown before and after medical intervention with (a) all patients, (b) cancer patients with surgery, (c) cancer patients with radiochemotherapy or (d) non-cancer patients with surgery and pharmacotherapy. McNemar’s test was used to analyze data. **P<0.01, *P<0.05.

As shown in Fig. 4A, the incidence rates of the following adverse events of grade ≥2 observed in all patients were significantly reduced after implementation of medical interventions listed in Table 1: insomnia (10.1% vs. 1.6%, P<0.01 by McNemar’s test), constipation (9.9% vs. 1.0%, P<0.01), nausea (8.0% vs. 0.9%, P<0.01), infection (7.7% vs. 0.7%, P<0.01), non-cancer pain (6.6% vs 2.5%, P<0.001), electrolytes (5.4% vs 0.4%, P<0.001), mucositis oral (5.0% vs. 1.2%, P<0.01), odynophagia (4.6% vs 2.6%, P = 0.004), neutrophil count decreased (4.5% vs 0.5%, P<0.01) and tumor pain (4.0% vs 1.2%, P<0.001). Similar data were obtained in subgroups such as cancer patients with surgery, those receiving chemoradiotherapy and non-cancer patients (Fig. 4B–D).

### Risks for prolongation of hospital stay among grade ≥2 adverse events

**Figure 5:**
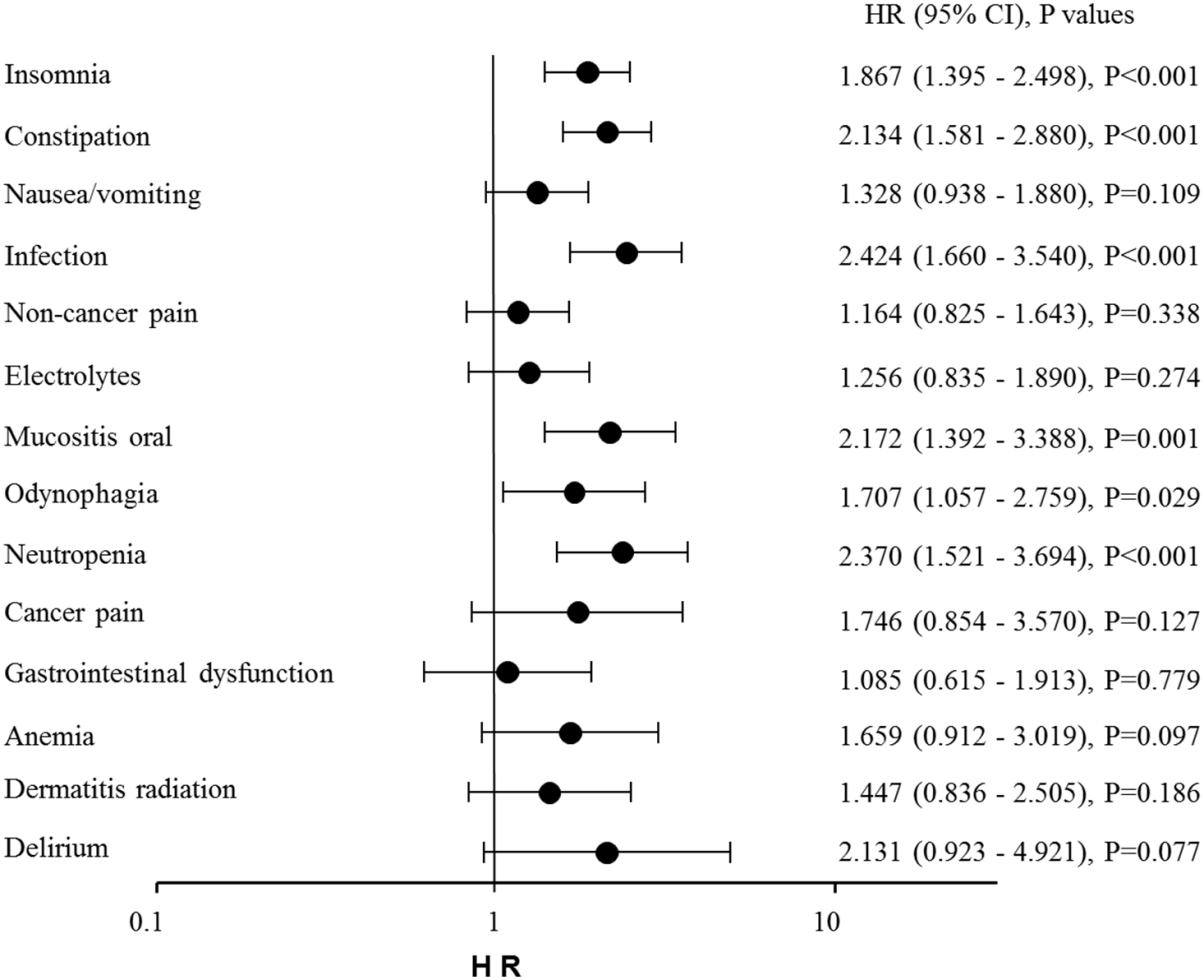
Multivariate Cox proportional hazard analysis of prolongation of hospital stay associated with grade ≥2 adverse events.

Univariate Cox proportional hazard analysis showed that grade ≥2 adverse events, including insomnia, constipation, nausea/vomiting, non-cancer pain, odynophagia, infection, oral mucositis, neutropenia, cancer pain, delirium, abnormal electrolytes, anemia and diarrhea, were risk factors for prolongation of hospital stay (data not shown). Subsequent multivariate Cox proportional hazard analysis revealed that the following six adverse events were found to be significant risks for prolongation of hospital stay: insomnia (1.867, 1.395–2.498, P<0.001), constipation (2.134, 1.581–2.880, P<0.001), infection (2.424, 1.660–3.540, P<0.001), oral mucositis (2.172, 1.392–3.388, P = 0.001), odynophagia (1.707, 1.057–2.759, P = 0.029) and neutropenia (2.370, 1.521–3.694, P<0.001) (Fig. 5).

## Discussion

In the present study, 41.3% (236/571) of patients who hospitalized in otolaryngology ward suffered from head and neck cancer. Adverse events of grade ≥2 occurred at high frequency in these patients, particularly in those receiving chemoradiotherapy, in which the incidence rates were over 20% in nausea/vomiting, constipation, oral mucositis, neutropenia and odynophagia. Infection occurred frequently in cancer patients with surgery, which was caused mainly by surgical site infection or aspiration pneumonitis after surgery. On the other hand, constipation and insomnia appeared frequently in all subgroups. Medications for prevention of nausea/vomiting, oral mucositis, surgical site infection and constipation due to opioid analgesics were given, according to the clinical practice guidelines [12], [15], [16], [19]. Nevertheless, the incidence of adverse events was unexpectedly high, where approximately one-third of hospitalized patients experienced grade ≥2 adverse events.

Despite the exclusion of the above-mentioned preventable adverse events, the incidence of adverse events in the present study was higher than those reported in other studies. A retrospective review of 1,014 medical and nursing records from two British hospitals reported an overall rate of adverse events of 10.8% [1]. The Canadian Adverse Events Study indicated that the overall incidence rate of adverse events for hospitalized patients is 7.5% [2]. A retrospective study of a stratified random sample of 10 hospitals in the United States identified 588 adverse events (25.1%) in 2,341 admissions [3]. The difference in the incidence of adverse events may be due to the difference in the patient population and the severity of adverse events. In our study, adverse events were graded according to CTCAE version 4.0 and the incidence of grade ≥2 events was evaluated, whereas the adverse events reported by other investigators were severe events that prolonged hospital stay and/or those required intervention. In particular, critically ill patients are at increased risk of adverse events. Parmentier-Decrucq et al. [4] reported by a prospective, observational study in critically ill patients that 120 of 262 transports (45.8%) were associated with adverse events.

Adverse events affect prognosis of a variety of diseases, increase the length of hospital stay and mortality, and the resultant medical costs are heightened. Classen et al. [7] reported that adverse events cause a significant prolongation of hospital stay, while other studies showed that adverse events are associated with increased time to hospital discharge [8]. Particularly, the cost for hospitalization in patients showing adverse events in the intensive care unit was substantial [9].

In our study, the length of hospital stay was dependent on the severity of adverse events, as evidenced by the increase in the mean duration of hospital stay from 9.2 days (grade 0 events) to 47.0 days (grade ≥3 events). Such adverse events-associated prolongation of hospital stay was observed not only in cancer patients receiving chemoradiotherapy but also in those with surgery and in non-cancer patients, thereby suggesting that adverse events increases the length of hospital stay regardless of the disease condition.

On the other hand, the length of hospital stay of patients showing improvement of grade ≥2 adverse events after medical intervention was significantly shorter than those who did not. This indicated that medical intervention is highly effective in reducing the length of hospital stay for patients who experienced moderate to severe adverse events. Medical intervention was also beneficial from a healthcare economic point of view: the cost saving due to reduction of hospital stay during the study period was estimated to be 53.0 million Japanese yen (USD 517,000) during 18 months.

A multivariate Cox proportional hazard analysis showed that several adverse events, including constipation, insomnia, infection, oral mucositis, odynophagia and neutropenia, were associated with significant risks for prolongation of hospital stay. Several investigators have reported that the incidence of infection, oral mucositis, and odynophagia, all of which were caused mainly by radiotherapy, is associated with longer hospital stay [20], [21]. It has been reported that neutropenia has been reported to be one of causes of prolonged hospitalization due to the development of febrile neutropenia [22].

On the other hand, we could not explain why constipation and insomnia caused a prolongation of hospital stay in the present study. A possibility that such adverse events may develop as a result of prolonged hospital stay could not be ruled out.

The incidence of a variety of adverse events, including those of high-risk of prolonged hospitalization, was significantly lowered by implementation of medical intervention, which may contribute at least in part to the reduction in hospital stay. Among various adverse events, odynophagia is a common and serious problem in patients receiving chemoradiotherapy for the therapy of head and neck cancer [23], [24]. It has also been shown that dysphagia is associated with lower survival rates [23]. Improvement in odynophagia may shorten hospital stay in patients with head and neck cancer, although no effective treatment for odynophagia has been confirmed at present. Intensity-modulated radiotherapy [25] and swallowing exercise [26] may reduce the severity of odynophagia.

On the other hand, it seems to be more likely that the prevention rather than relief of adverse events is important in reducing the length of hospital stay. We previously reported that polaprezinc, a zinc-containing anti-ulcer agent, prevents oral mucositis associated with chemoradiotherapy, in which the incidence of grade ≥2 events was significantly (P = 0.009) lower in polaprezinc-treated group (40.0%) than in azulene gargle-treated control group (86.7%) [27]. In the present study, all 121 patients who underwent chemoradiotherapy for head and neck cancer were pretreated with polaprezinc for prevention of oral mucositis, and the incidence of grade ≥2 symptom was 21.5% (26 of 121 patients), indicating that the occurrence of moderate to severe oral mucositis was markedly reduced by such premedication in several patients.

Several limitations exist in the present study. First, this was a non-randomized single center study. Second, the study was carried out in a ward (otolaryngology ward) of a medium size university hospital. Thus, the sample size was small and the patient population was limited to the surgical field. Third, the majority of adverse events observed in the present study resulted from chemoradiotherapy for head and neck cancer. Fourth, our study focused on the effect of pharmacological management of adverse events on the length of hospitalization. Thus, the contribution of some risk factors other than adverse events to the prolongation of hospital stay was not considered. Finally, the medical expense was confined to the hospital charge. Other factors such as cost of drugs for the treatment of adverse events were not counted in the present study. Further studies in multiple wards of a number of institutions are required to demonstrate the influence of adverse events on the duration of hospital stay and the effectiveness of medical intervention from the viewpoints of safety and medical economics.

## Conclusions

In conclusion, we investigated the incidence of adverse events in all patients who admitted to the otolaryngology ward of our hospital during a period of 18 months, and found that grade ≥2 adverse events occurred in approximately one third of all patients. It was noteworthy that the incidence of adverse events caused a prolongation of hospital stay, in which the length of hospitalization increased in a manner dependent on the grade of adverse events. Multivariate Cox proportional hazard analysis revealed that several adverse events, including constipation, insomnia, oral mucositis, odynophagia, infection, and neutropenia, were significant risks for prolongation of hospital stay. Implementation of medical intervention to relief the symptoms of adverse events was highly effective in reducing the duration of hospital stay and the resultant medical cost.
